# Mitochondrial variants of complex I genes associated with leprosy clinical subtypes

**DOI:** 10.1038/s41598-024-57191-y

**Published:** 2024-03-16

**Authors:** Felipe Gouvea de Souza, Caio S. Silva, Gilderlanio S. de Araújo, Mayara N. Santana-da-Silva, Angélica Rita Gobbo, Moisés Batista da Silva, Pablo Pinto, Patrícia Fagundes da Costa, Claudio Guedes Salgado, Ândrea Ribeiro-dos-Santos, Giovanna C. Cavalcante

**Affiliations:** 1 Laboratório de Genética Humana e Médica, Instituto de Ciências Biológicas, Universidade Federal do Pará, Belém, PA 66075-110 Brazil; 2 Laboratório de Dermato-Imunologia, Instituto de Ciências Biológicas, Universidade Federal do Pará, Marituba, PA 67105-290 Brazil

**Keywords:** Leprosy, Mitochondria, mtDNA, Complex I, Heteroplasmy, Medical genomics, Infectious diseases

## Abstract

Leprosy is a chronic bacterial infection mainly caused by *Mycobacterium leprae* that primarily affects skin and peripheral nerves. Due to its ability to absorb carbon from the host cell, the bacillus became dependent on energy production, mainly through oxidative phosphorylation. In fact, variations in genes of Complex I of oxidative phosphorylation encoded by mtDNA have been associated with several diseases in humans, including bacterial infections, which are possible influencers in the host response to leprosy. Here, we investigated the presence of variants in the mtDNA genes encoding Complex I regarding leprosy, as well as the analysis of their pathogenicity in the studied cohort. We found an association of 74 mitochondrial variants with either of the polar forms, Pole T (Borderline Tuberculoid) or Pole L (Borderline Lepromatous and Lepromatous) of leprosy. Notably, six variants were exclusively found in both clinical poles of leprosy, including m.4158A>G and m.4248T>C in *MT-ND1*, m.13650C>A, m.13674T>C, m.12705C>T and m.13263A>G in *MT-ND5,* of which there are no previous reports in the global literature. Our observations reveal a substantial number of mutations among different groups of leprosy, highlighting a diverse range of consequences associated with mutations in genes across these groups. Furthermore, we suggest that the six specific variants exclusively identified in the case group could potentially play a crucial role in leprosy susceptibility and its clinical differentiation. These variants are believed to contribute to the instability and dysregulation of oxidative phosphorylation during the infection, further emphasizing their significance.

## Introduction

Leprosy is one of the oldest known human infectious diseases^1^. Primarily caused by the infectious agent *Mycobacterium leprae*, but also caused by the infectious agent *M. lepromatosis*, this chronic granulomatous bacterial disease presents symptoms that mainly involve the skin, the peripheral nervous system and the reticuloendothelial system, but other systems, such as the upper respiratory tract, bones and joints, eyes and adrenal glands can also be affected^1–4^. The Ridley–Jopling system classifies leprosy into polar groups: Lepromatous (LL) and Tuberculoid (TT); and unstable borderline spectrum: Borderline Tuberculoid (BT), Borderline Borderline (BB) and Borderline Lepromatous (BL)^1,5^.

Notably, *M. leprae* is dependent on the production of energy and nutritional products by the host, resulting from the host–pathogen interaction, undoubtedly involving the main function of mitochondria of energy generation, due to the cellular signaling pathways in which these organelles participate and connect their metabolism to meet their nutrient demands^6,7^. Furthermore, due to the decay of the *M. leprae* genome, which leads to strain uniformity, it is suggested that the genetic background of the host, and not bacterial variability, is a central aspect of susceptibility to leprosy^1^.

In this sense, mitochondria play a key role in the production of energy in the form of Adenosine Triphosphate (ATP), both by the tricarboxylic acid cycle (TCA) and mainly by oxidative phosphorylation (OXPHOS), in which the electrons produced in the TCA are pumped from the mitochondrial matrix into the intermembrane space (IMS) and transferred through the respiratory complexes in the electron transport chain (ETC)^8–12^. The ETC consists of five protein complexes (I–V) and two electron carriers (ubiquinone and cytochrome *c*), with Complex I being the main entrance and the largest component of this respiratory chain and of crucial importance in cell metabolism for reducing quinone by oxidation of NADH and regeneration of NAD+ (nicotinamide adenine dinucleotide, oxidized) in the mitochondrial matrix^10,12–14^.

Abnormalities in the formation and function of OXPHOS complexes are associated with genetic mutations and can lead to various defects, including synapse damage, axonal degeneration, excessive ROS production, apoptosis and cell death, influencing the process of complex diseases, such as cancer, neurodegenerative diseases and infectious diseases^6,10,15^.

In fact, variations specifically in Complex I genes encoded by mtDNA have been associated with several diseases in humans, such as metabolic diseases, neurodegenerative disorders and bacterial infections^14,16^. Furthermore, it is important to mention that different cells have a variable number of mtDNA copies, with the presence of two or more genotypes of the same mutation in varying proportions^15,17^. This phenomenon is called mitochondrial heteroplasmy and, despite being a normal part of healthy human biology, it is also relevant in pathological processes^18,19^.

Importantly, these mutations and their levels of heteroplasmy may influence the host response to leprosy, leading to differences in the evolution of this infection. In this study, we investigated the presence of variants in mtDNA genes encoding Complex I regarding leprosy, in search of new potential biomarkers of development and progression of this disease, while also assessing the nuclear and mitochondrial genomic ancestry of our cohort.

## Results

### Genomic ancestry

**Figure 1:**
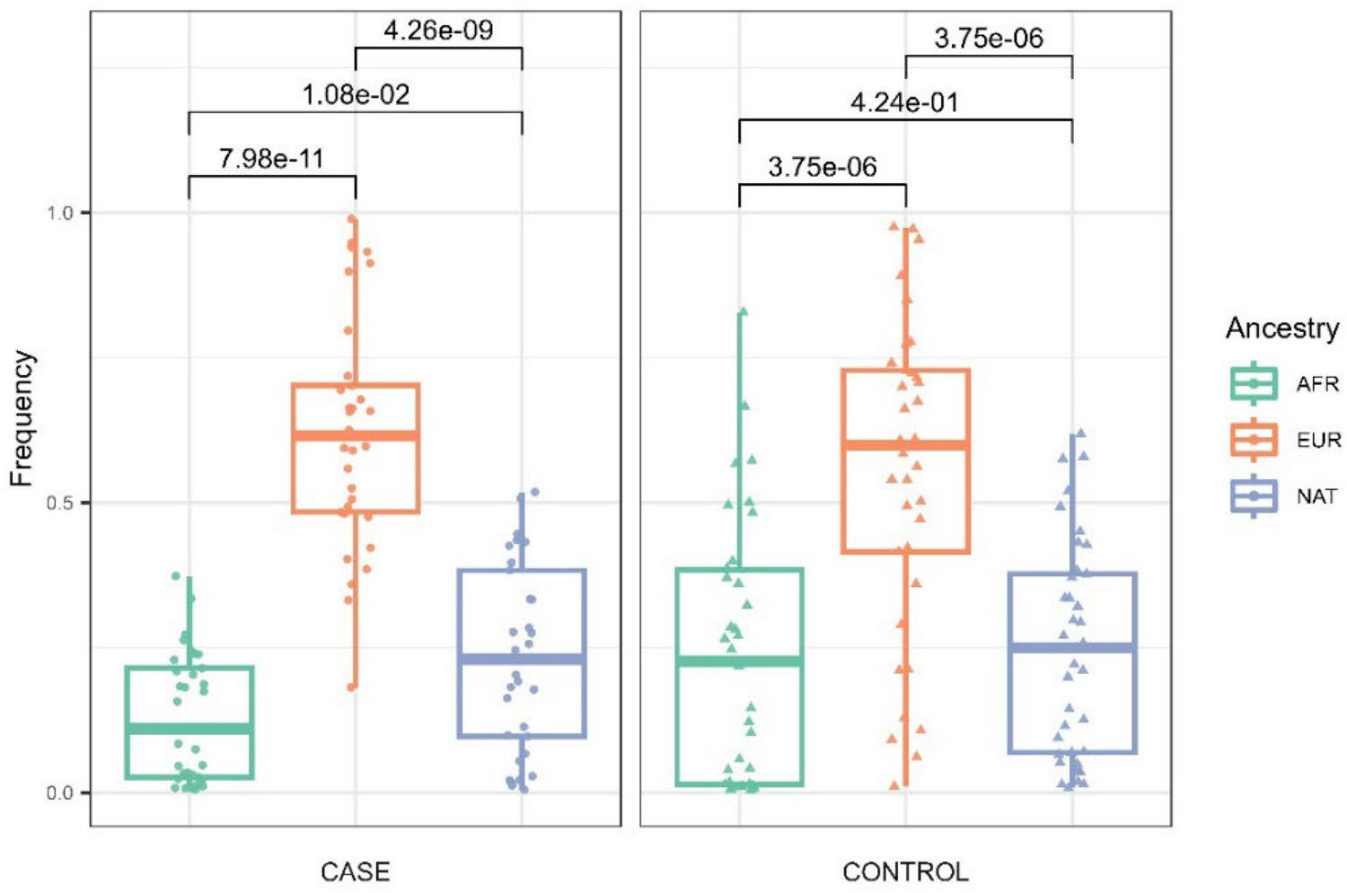
Distribution of the three nuclear ancestries (African-AFR, European-EUR and Native American-NAT) was found in both case and control groups.

It is widely known that the Brazilian population is ethnically diverse, so we analyzed the genomic ancestry composition of our cohort through a set of nuclear markers previously developed by our research group^20,21^. We observed a similar pattern of nuclear genomic ancestry between case and control (Fig. 1), with European contribution more present in both groups (EUR; case mean = 0.613; control mean = 0.558), followed by Native American (NAT; case mean = 0.234; control mean = 0.246) and African (AFR; case mean = 0.136; control mean = 0.236) contributions. When comparing the paired case and control ancestries, there was no significant difference between the groups; however, when the ancestries were compared to each other in the case group, all of them were statistically significant (Mann–Whitney test, NAT vs. EUR, p-value = 4.26e−09; NAT vs. AFR, p-value = 1.08e−02; EUR vs. AFR, p-value = 7.98e−11), which demonstrates heterogeneity in this sample group. In addition, although our cohort is made up of an intrinsically admixed population, both cases and controls present similarly distributed ancestry, making this a good endemic control for further analysis. Recently, we demonstrated that, by analyzing the mitochondrial ancestry in the same cohort, the European haplogroup H2 was the most frequent in both groups; however, the Native American haplogroups A, B, C and D together accounted for nearly half of the maternal ancestry contribution^22^.

**Figure 2:**
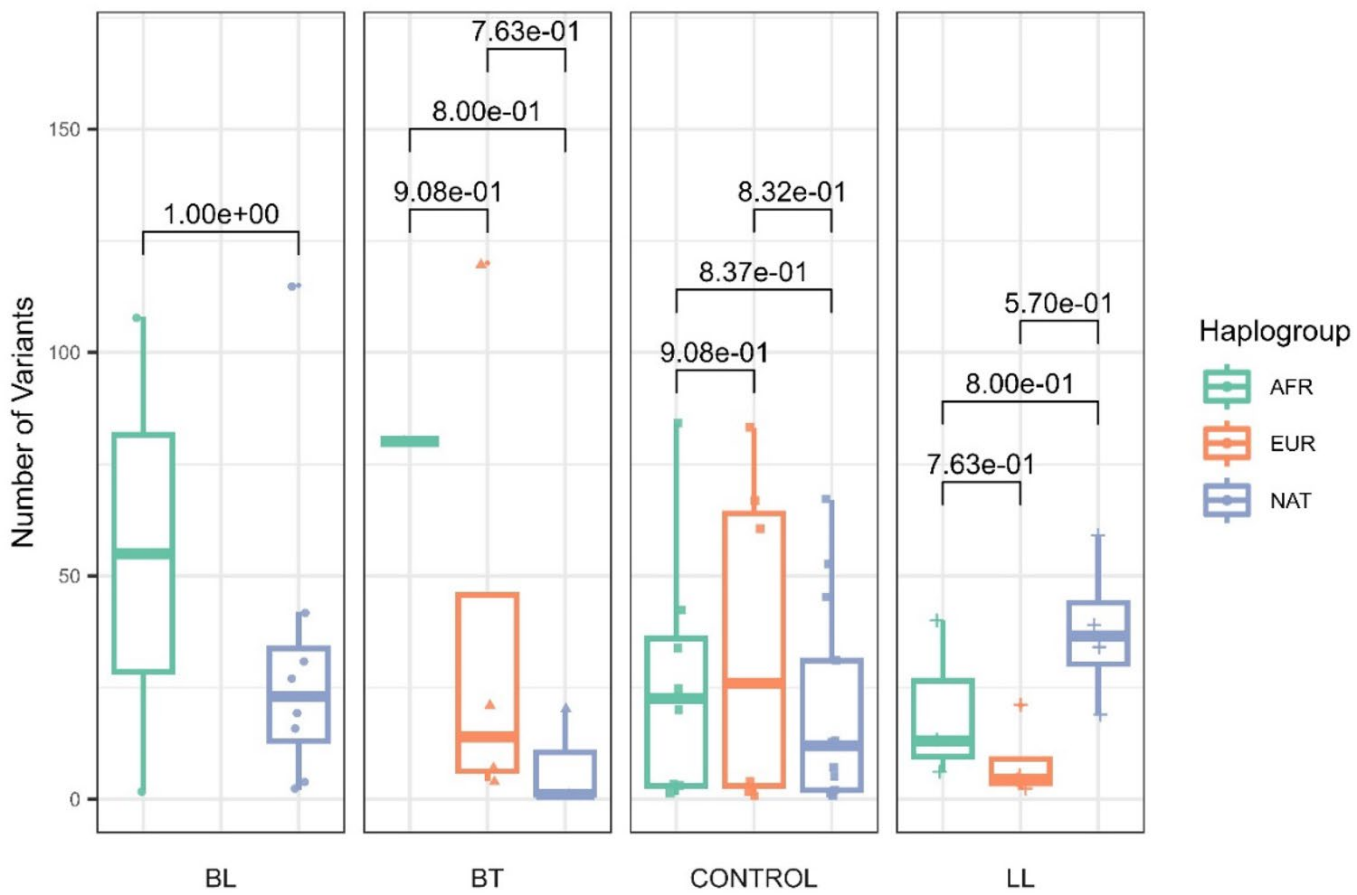
Number of Complex I variants in the three ancestries (African-AFR, European-EUR, Native American-NAT) in each leprosy subtype (BL, BT, and LL) and control group.

Then, considering the mitochondrial ancestry, we analyzed the distribution of mitochondrial variants of Complex I genes in the three leprosy subtypes and the control group, filtering the mutations for those with heteroplasmy > 0.05 and < 0.95 (Fig. 2). It is possible to observe a different pattern between the number of heteroplasmic variants among leprosy subtypes. Despite European ancestry being the most frequent in both groups, it did not correlate with the proportion of heteroplasmic variants between the subtypes and the control group.

**Figure 3:**
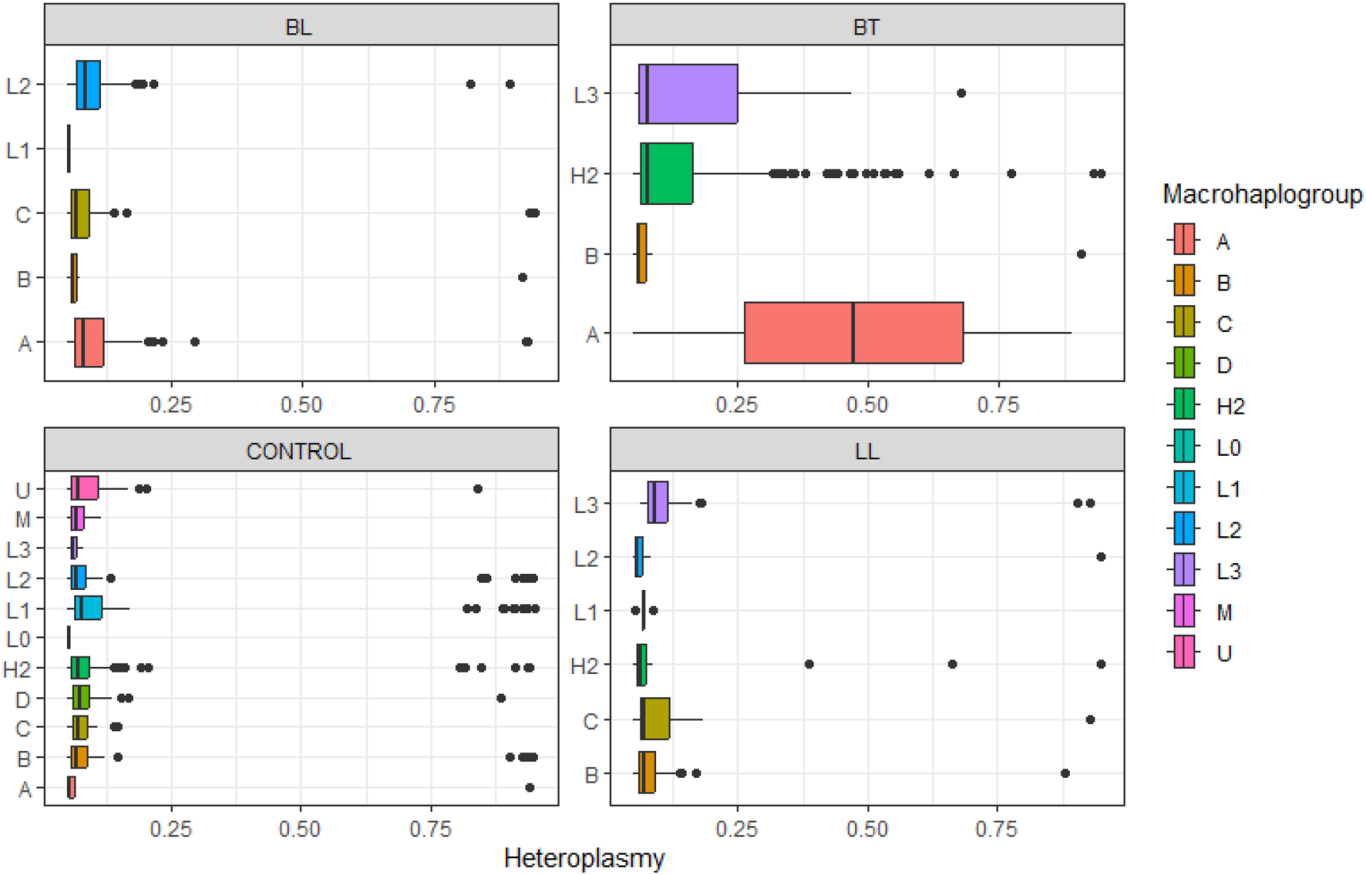
Heteroplasmy levels of variants according to each macrohaplogroups in the control group and the case subgroups (BT, BL, and LL).

Finally, we stratified the macrohaplogroups belonging to the control group and the case subtypes (BT, BL, LL) and analyzed the levels of heteroplasmy of the variants in both groups according to each macrohaplogroup (Fig. 3). After this processes, it was possible to observe that the BT subgroup presented a higher level of heteroplasmy in the macrohaplogroups A and L3 that belong to Native American and African ancestry, respectively, in addition to presenting a more prominent heteroplasmic profile than the BL and LL subgroups, which, in turn, presented a slightly more similar pattern to each other. This heterogeneity between the subgroups suggests that the levels of mitochondrial heteroplasmy may differ depending on the polarization of the clinical forms of leprosy, thus being able to influence the oxidative process and the development of the disease.

### Distribution and characterization of mitochondrial variants

**Figure 4:**
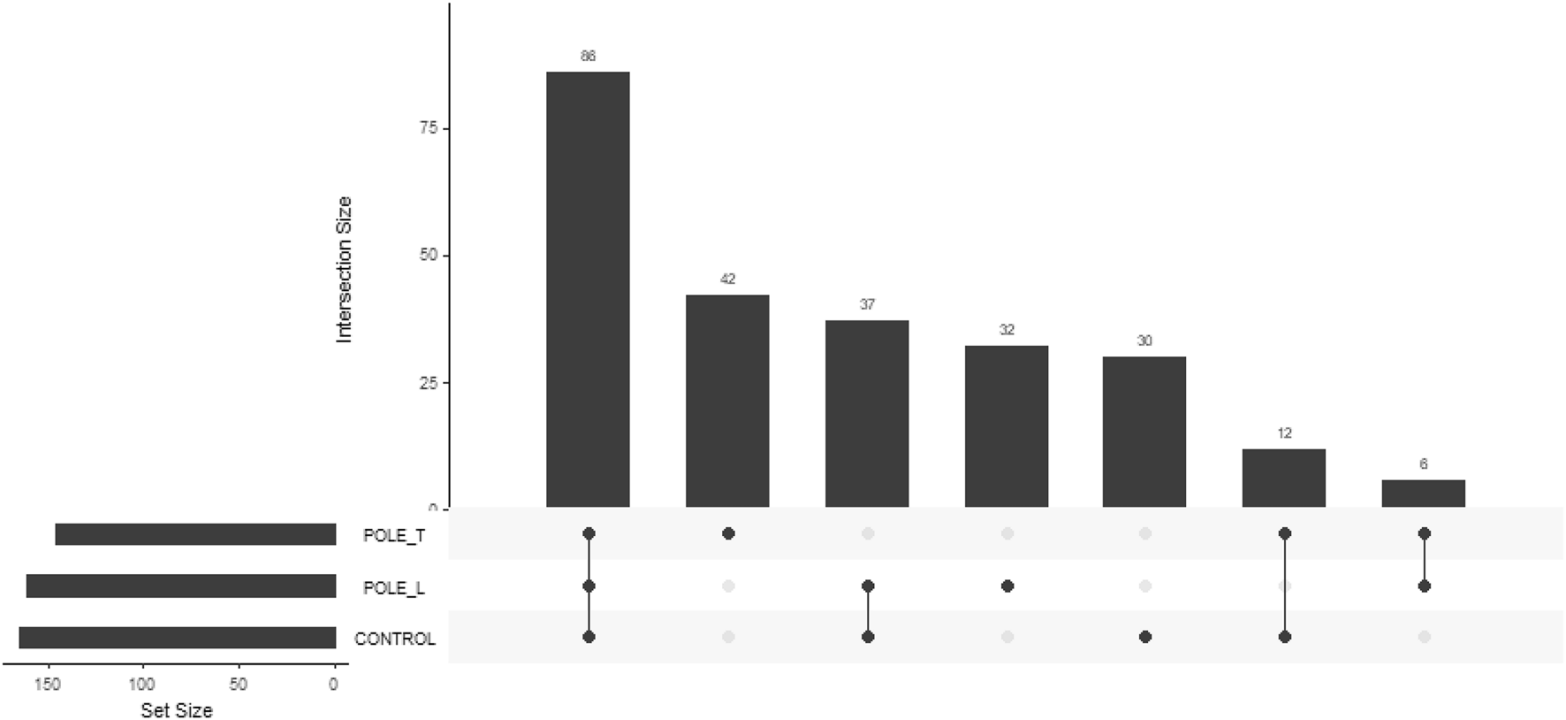
Distribution of found mitochondrial variants in the healthy control group and the leprosy poles (Pole T, and Pole L). Each dark dot indicates the group with the respective number of variants and each line represents the intersection between groups. The set size is the overall number of variants.

Furthermore, we analyzed the number of variants that were exclusive to the leprosy poles (Pole L being constituted by subtypes BL and LL; Pole T consisting of subtype BT) and to control, as well as those shared between such groups, that is, the intersection between groups, according to the heteroplasmy filtering of > 0.05 and < 0.95 (Fig. 4). Interestingly, the control contains the higher number of exclusive variants (n = 86), which might be protective against leprosy development, and 74 variants were exclusive of only one leprosy pole (either Pole T or Pole L), suggesting that these variants might influence the onset of specific clinical forms of leprosy.

**Figure 5:**
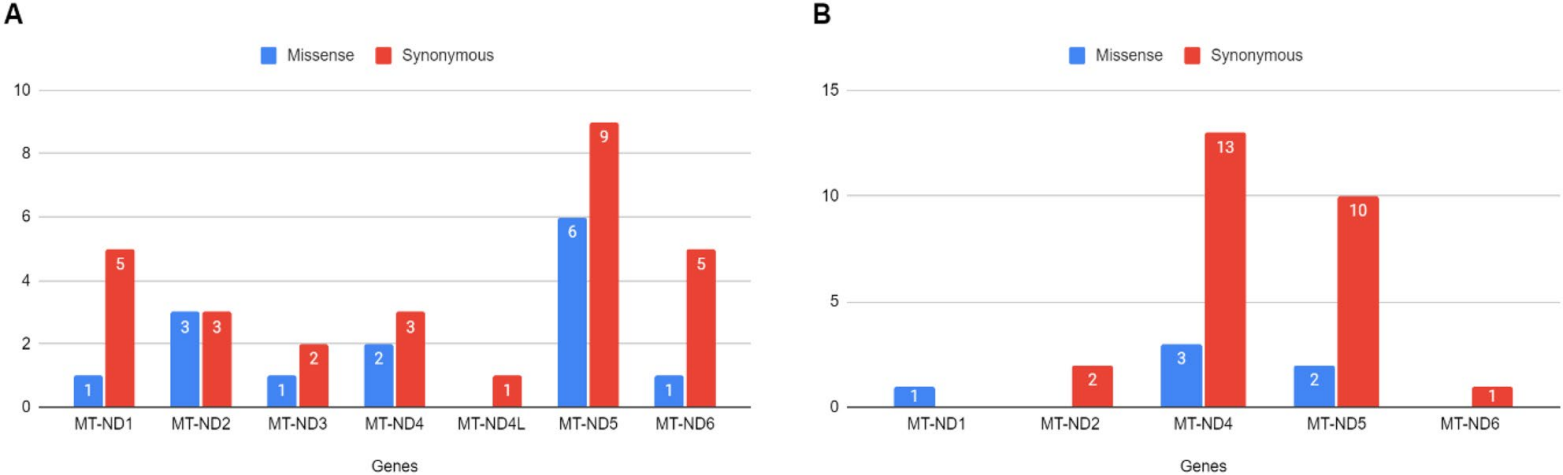
Distribution of shared variants in leprosy poles by gene with their predicted consequence. (**A**) Variants found only in the leprosy Pole T. (**B**) Variants found only in the leprosy Pole L.

Remarkably, when analyzing the predictions of the pathogenicity of the mutations exclusive to one of the poles (Pole T = 42, Pole L = 32), it is possible to observe a predominance of synonymous mutations in leprosy poles (Fig. 5). It is interesting to note that there is a more generalized distribution of mutations by genes in the pole T, with *MT-ND1*, *MT-ND5* and *MT-ND6* presenting the greatest number of variants (Fig. 5A). Furthermore, when analyzing the mutations per gene, *MT-ND4* and *MT-ND5* stand out for having a greater number of variants in pole L when compared to the other analyzed Complex I genes, indicating that mutations in these genes may particularly influence susceptibility to this disease (Fig. 5B).

Notably, six variants were shared between both clinical poles of leprosy, but not by the control (Fig. 4), suggesting these variants might play a role in leprosy development, regardless of the subtype. The pathogenicity characterization of these variants is shown in Table 1. Five variants were characterized as synonyms and one as missense (*MT-ND1*).

**Table 1:** In silico pathogenicity characterization of the variants found only in the leprosy poles (Pole T and Pole L).

Genes	Mutation	Consequence	dbSNP	ClinVar
*MT-ND1*	m.4158A>G	Synonymous variant	rs1603219327	Benign
	m.4248T>C	Missense variant	rs9326618	Benign
*MT-ND5*	m.13650C>A	Synonymous variant		
	m.13674T>C	Synonymous variant	rs1603224299	
	m.12705C>T	Synonymous variant	rs193302956	
	m.13263A>G	Synonymous variant	rs28359175	

## Discussion

Although leprosy is considered a relevant public health problem in multiple countries, it is still understudied and neglected, especially concerning mitochondrial genetic aspects and, particularly, in the northern region of Brazil^22,23^. The Brazilian population is highly mixed, with a genetic contribution from different ethnic groups, mainly European, Native American and African groups^24,25^. In this perspective, the introduction of leprosy in Brazil may be associated with the period of colonization of the country by the Portuguese and, to a lesser extent, by the Dutch, French and Spanish, as well as the slave trade from Africa^26–28^.

Extensive research indicates with a high level of confidence that the ancestral genetic composition significantly influences the susceptibility and/or resistance to various diseases, including cancer and diabetes, and it is strongly associated with the immune response to infectious diseases, including leprosy^6,8,26^. In addition, in case–control association studies, genetic ancestry is of great relevance in mixed populations, given that this genetic diversity can influence genotypic distribution and phenotypic heterogeneity due to population stratification^24^.

Importantly, our findings on nuclear and mitochondrial ancestry corroborate the historical processes of colonization and population formation in northern Brazil, with a prevalence of European and Native American ancestry in the studied group (Fig. 1)^22,29^. Da Silva et al*.* demonstrated in their study that the contribution of different ethnic groups in the genetic composition of the Amazonian population can influence the risk of developing leprosy, with European ancestry having a great influence on this susceptibility^24^. Furthermore, Pinto et al*.*, when analyzing different INDEL markers for nuclear ancestry, indicated that an increase in the European contribution to the population increases the risk of developing leprosy, while an increase in the African contribution decreases the risk of developing leprosy, and the analysis of Native American contribution did not result in any statistically significant difference^27^.

However, mitochondrial ancestry is still an interesting matter to be debated, as there are still few studies on whether mitochondrial haplogroups can confer genetic susceptibility to mycobacterial infection. One of the only studies in this regard showed that, in the Han Chinese population, haplogroups did not confer susceptibility to leprosy^6^. However, in our study, it was possible to observe that the Native American macrohaplogroup was more prevalent in all subtypes of the disease, which suggests that this ancestry may influence the development of distinct leprosy subtypes, particularly BL after *M. leprae* infection^22^. Furthermore, the different profiles of heteroplasmy found in our study between mitochondrial genes in the case subgroups (especially BT) and the control group suggest that heteroplasmy can influence the dysregulation of mitochondrial oxidative processes and the type of host immune response to *M. leprae*, dividing them into the clinical forms of leprosy^22,30–32^.

Regarding Complex I of OXPHOS, Tió-Coma et al. analyzed gene expression by RNA sequencing in blood samples between patients before the presence of leprosy symptoms and contact control patients, as well as differential longitudinal gene expression between each patient. Among the results of that study, five mitochondrial genes (*MT-ND2*, *MT-ND4*, *MT-ND5*, *MT-CO1* and *MT-CYB*) were found involved in OXPHOS and negatively regulated in leprosy patients, thus presenting a disadvantage for the successful elimination of *M. leprae*^33^. It is worth noting that three of these genes (*MT-ND2*, *MT-ND4*, *MT-ND5*) encode Complex I, with *MT-ND2* and *MT-ND6* being essential subunits for the formation of the NADH dehydrogenase respiratory chain in the mitochondrial membrane, playing a critical role in oxidative phosphorylation^33^.

It is necessary to highlight that several studies indicate that variants in mtDNA genes are involved in various infectious processes, cancer and neurodegenerative diseases^9,34^. In the study by Tonsing et al*.*, the authors analyzed the entire mitochondrial genome of blood samples from patients with tuberculosis to understand the mtDNA variants that may predispose the analyzed population to the disease, and observed a total of 83 variants in non-coding regions, in addition to two mutations with deleterious/damaging effects on *MT-ND2* genes (m.4824G>A) and *MT-ND6* (m.14180C>T)^35^. In addition, these variants are close to a highly conserved position of the protein, which can cause an empty space inside the molecule, breaking hydrogen bonds and disturbing the correct folding of the protein^35^. In our study, we found the same mutations, with the m.4824G>A observed in two patients with the BT subtype and one in the control group, which may indicate that, in the case of *M. leprae* infection, that mutation could influence susceptibility to the disease due to its prevalence in the case group.

Importantly, as seen in Table 1, one of the variants that were shared between all leprosy poles, but not found in the control, is a missense variant with a benign prediction. The other five variants are synonymous, and they are present in *MT-ND5*. Although most of these variants have identification in the dbSNP, as far as we know, there are no previous reports in the global literature on these six variants, nor reports on the ClinVar database in association with other diseases.

Here, we investigated variants in mitochondrial genes encoding Complex I subunits in leprosy patients and contact controls from a Brazilian population. It was possible to observe a significant number of shared mutations by all groups and mutations present only in the case group, suggesting that mitochondrial dysfunction, highlighted by the energy generation of OXPHOS, is implicated in the pathogenesis of leprosy and the viability of *M. leprae* in the host.

In addition, in the analyses of the genomic ancestry of this population, it was possible to observe that the mitochondrial haplogroups, mainly the European and African haplogroups, could influence the increased risk or protection against leprosy, respectively. This is particularly important because, to date, there are no studies in the literature that associate mitochondrial ancestry with the development of leprosy. Thus, more clinical studies with larger cohorts should be carried out, using robust statistical tests, to investigate the mitochondrial genetic profile of individuals affected by the disease and the influence of the mitochondrial genes that encode OXPHOS complexes, to expand the understanding of the pathogenesis of *M. leprae* and suggest potential biomarkers for early detection of leprosy.

## Methods

### Sampling

Blood samples were obtained from patients affected by leprosy (case group), composed of borderline lepromatous (BL) leprosy (*n* = 12), lepromatous (LL) leprosy (*n* = 11) and borderline tuberculoid (BT) leprosy (*n* = 10), in addition to unrelated household individuals (*n* = 37, control group), all residents of Pará state, Brazil. For each patient, both case and control groups, one blood sample (5 mL) was collected. The clinical and laboratory diagnosis of the patients was established by the parameters previously described^5^. This study adhered to the Declaration of Helsinki and was approved by the Ethics Committee of the Institute of Health Sciences at the Federal University of Pará (CEP-ICS/UFPA n. 197/07), and all participants read and signed an informed consent form.

### DNA extraction and quantification

Total DNA was extracted by phenol–chloroform method based on Sambrook et al.^36^. Quantification to verify the concentration and purity of the extracted material was performed with NanoDrop 1000 spectrophotometer (Thermo Fisher Scientific, Wilmington, DE, USA).

### Amplification and sequencing

Amplification of mtDNA from the total DNA was performed by conventional PCR with specific primers, as described by Cavalcante et al*.*^8^, to cover the entire mitochondrial genome. To verify the quality of the amplification, the samples were applied to a 1% agarose gel and, later, measured in a Qubit 2.0 fluorometer for the library preparation (Thermo Fisher Scientific).

The sequencing of the complete mitochondrial genome was previously described by our research group^22^. All samples (*n* = 70) were qualified for the whole mitochondrial genome sequencing. Nextera XT DNA Library Preparation Kit (Illumina Inc., Chicago, IL, USA) was used to prepare the libraries and MiSeq Reagent Kit V3 (600-cycles) (Illumina) for sequencing on the MiSeq System (Illumina), according to the manufacturer's instructions. During the preparation of the libraries, High Sensitivity D1000 ScreenTape was used on the Agilent 2200 TapeStation System (Agilent Technologies, Santa Clara, CA, USA) to assess the quality of the genetic material.

### Bioinformatics and statistical analyses

After sequencing, we updated the pipeline for bioinformatics analysis previously described^8^. Paired-end sequencing reads were trimmed to remove leading and trailing low quality and to scan reads with a 3-base wide sliding window. After trimming, the reads were aligned with the human reference mtDNA sequence (Revised Cambridge Reference Sequence, rCRS) and used to map and classify sequences. For the SNP calling, annotation and heteroplasmy detection, we used mutserve for each sample with the quality parameters as previously described^22^. Finally, for this study, we selected only the seven mitochondrial genes that encode Complex I proteins to further investigate the mutation profile: *MT-ND1*, *MT-ND2*, *MT-ND3*, *MT-ND4*, *MT-ND4L*, *MT-ND5* and *MT-ND6*.

The analysis of the prediction of the pathogenicity of the mutations was performed using Mitochondrial Disease Sequence Data Resource (MSeqDR, https://mseqdr.org/index.php)^37^, the Genome Aggregation Database (gnomAD, https://gnomad.broadinstitute.org/)^38^, the Single Nucleotide Polymorphism database (dbSNP, https://www.ncbi.nlm.nih.gov/snp/)^39^ and the ClinVar database (https://www.ncbi.nlm.nih.gov/clinvar/)^40^.

To analyze the genomic consequence, impact, and pathogenicity prediction of these variants, MSeqDR was used. As a double check and for lack of information on the variants on the platform described above, they were also sought on the gnomAD and dbSNP platforms. Finally, once the prediction of mutations was inferred, their clinical consequences were analyzed based on the data currently available at ClinVar.

All statistical analyses were performed in R (R Core Team)^41^ or JASP (JASP Team, 2022)^42^. R packages ggplot2^43^ and UpSetR^44^ were used for graphic representations. In all analyses, the p-value was considered statistically significant when ≤ 0.05.

### Ethics approval and consent to participate

The study was conducted in accordance with the Declaration of Helsinki and approved by the Ethics Committee of Institute of Health Sciences at the Federal University of Pará (CEP-ICS/UFPA n. 197/07), and all participants read and signed an informed consent form.

### Inclusion and diversity

We support inclusive, diverse, and equitable conduct of research.

## Data Availability

All raw sequences were previously deposited by Souza et al. (2023) at the European Nucleotide Archive (ENA), under accession number PRJEB59275.

## References

[1] Fava VM, Dallmann-Sauer M, Schurr E (2020). Genetics of leprosy: Today and beyond. Hum. Genet..

[2] Eichelmann K, González González SE, Salas-Alanis JC, Ocampo-Candiani J (2013). Leprosy. An update: Definition, pathogenesis, classification, diagnosis, and treatment. Actas Dermo-Sifiliográficas (English Edition).

[3] Maymone MBC, Laughter M, Venkatesh S, Dacso MM, Rao PN, Stryjewska BM, Hugh J, Dellavalle RP, Dunnick CA (2020). Leprosy: Clinical aspects and diagnostic techniques. J. Am. Acad. Dermatol..

[4] Han XY, Jessurun J (2013). Severe leprosy reactions due to *Mycobacterium*
*lepromatosis*. Am. J. Med. Sci..

[5] Ridley DS, Jopling WH (1966). Classification of leprosy according to immunity. A five-group system. Int. J. Lepr. Other Mycobact. Dis..

[6] Wang D, Su LY, Zhang AM, Li YY, Li XA, Chen LL, Long H, Yao YG (2012). Mitochondrial DNA copy number, but not haplogroup, confers a genetic susceptibility to leprosy in Han Chinese from Southwest China. PLoS One.

[7] de Souza FG, Cavalcante GC (2022). Mitochondria in *Mycobacterium* infection: From the immune system to mitochondrial haplogroups. IJMS.

[8] Cavalcante GC, Marinho ANR, Anaissi AK, Vinasco-Sandoval T, Ribeiro-dos-Santos A, Vidal AF, de Araújo GS, Demachki S, Ribeiro-dos-Santos Â (2019). Whole mitochondrial genome sequencing highlights mitochondrial impact in gastric cancer. Sci. Rep..

[9] Yan C, Duanmu X, Zeng L, Liu B, Song Z (2019). Mitochondrial DNA: Distribution, mutations, and elimination. Cells.

[10] Bergman O, Ben-Shachar D (2016). Mitochondrial oxidative phosphorylation system (OXPHOS) deficits in schizophrenia: Possible interactions with cellular processes. Can. J. Psychiatry.

[11] Liu S, Liu S, He B, Li L, Li L, Wang J, Cai T, Chen S, Jiang H (2021). OXPHOS deficiency activates global adaptation pathways to maintain mitochondrial membrane potential. EMBO Rep..

[12] Vercellino I, Sazanov LA (2022). The assembly, regulation and function of the mitochondrial respiratory chain. Nat. Rev. Mol. Cell Biol..

[13] Formosa LE, Ryan MT (2018). Mitochondrial OXPHOS complex assembly lines. Nat. Cell Biol..

[14] Hirose M, Schilf P, Zarse K, Busch H, Fuellen G, Jöhren O, Köhling R, König IR, Richer B, Rupp J (2019). Maternally inherited differences within mitochondrial complex I control murine healthspan. Genes.

[15] Andrieux P, Chevillard C, Cunha-Neto E, Nunes JPS (2021). Mitochondria as a cellular hub in infection and inflammation. IJMS.

[16] Ng YS, Thompson K, Loher D, Hopton S, Falkous G, Hardy SA, Schaefer AM, Shaunak S, Roberts ME, Lilleker JB (2020). Novel MT-ND gene variants causing adult-onset mitochondrial disease and isolated complex I deficiency. Front. Genet..

[17] Pérez-Amado CJ, Bazan-Cordoba A, Hidalgo-Miranda A, Jiménez-Morales S (2021). Mitochondrial heteroplasmy shifting as a potential biomarker of cancer progression. IJMS.

[18] Prates Mori M, de Souza-Pinto NC (2018). Role of mitochondrial dysfunction in the pathophysiology of DNA repair disorders: Mitochondrial role in DNA repair disorders. Cell Biol. Int..

[19] Roca-Bayerri C, Robertson F, Pyle A, Hudson G, Payne BAI (2021). Mitochondrial DNA damage and brain aging in human immunodeficiency virus. Clin. Infect. Dis..

[20] Santos NPC, Ribeiro-Rodrigues EM, Ribeiro-dos-Santos ÂKC, Pereira R, Gusmão L, Amorim A, Guerreiro JF, Zago MA, Matte C, Hutz MH (2010). Assessing individual interethnic admixture and population substructure using a 48-insertion-deletion (INSEL) ancestry-informative marker (AIM) panel. Hum. Mutat..

[21] de Ramos BRA, D’Elia MPB, Amador MAT, Santos NPC, Santos SEB, da Cruz Castelli E, Witkin SS, Miot HA, Miot LDB, da Silva MG (2016). Neither self-reported ethnicity nor declared family origin are reliable indicators of genomic ancestry. Genetica.

[22] De Souza FG, Da Silva MB, De Araújo GS, Silva CS, Pinheiro AHG, Cáceres-Durán MÁ, Santana-da-Silva MN, Pinto P, Gobbo AR, Da Costa PF (2023). Whole mitogenome sequencing uncovers a relation between mitochondrial heteroplasmy and leprosy severity. Hum. Genom..

[23] Soares AMM, da Corrêa RGCF, dos Santos KCB, Figueiredo IA, de Paiva MFL, de Aquino DMC (2021). Leprosy cases diagnosed by contacts examination in a hyperendemic capital city of northeastern Brazil. Anais Brasileiros de Dermatologia.

[24] da Silva MNS, da Veiga Borges Leal DF, Sena C, Pinto P, Gobbo AR, da Silva MB, Salgado CG, dos Santos NPC, dos Santos SEB (2022). Association between SNPs in microRNAs and microRNAs-machinery genes with susceptibility of leprosy in the amazon population. IJMS.

[25] Kehdy FSG, Gouveia MH, Machado M, Magalhães WCS, Horimoto AR, Horta BL, Moreira RG, Leal TP, Scliar MO, Soares-Souza GB (2015). Origin and dynamics of admixture in Brazilians and its effect on the pattern of deleterious mutations. Proc. Natl. Acad. Sci. USA.

[26] Cardona-Castro N, Cortés E, Beltrán C, Romero M, Badel-Mogollón JE, Bedoya G (2015). Human genetic ancestral composition correlates with the origin of *Mycobacterium*
*leprae* strains in a leprosy endemic population. PLoS Negl. Trop. Dis..

[27] Pinto P, Salgado C, Santos NPC, Santos S, Ribeiro-dos-Santos Â (2015). Influence of genetic ancestry on INDEL markers of NFKβ1, CASP8, PAR1, IL4 and CYP19A1 genes in leprosy patients. PLoS Negl. Trop. Dis..

[28] Cunha C, Pedrosa VL, Dias LC, Braga A, Chrusciak-Talhari A, Santos M, Penna GO, Talhari S, Talhari C (2015). A historical overview of leprosy epidemiology and control activities in Amazonas, Brazil. Rev. Soc. Bras. Med. Trop..

[29] Schaan AP, Costa L, Santos D, Modesto A, Amador M, Lopes C, Rabenhorst SH, Montenegro R, Souza BDA, Lopes T (2017). MtDNA structure: The women who formed the Brazilian Northeast. BMC Evol. Biol..

[30] Hudson G, Carelli V, Spruijt L, Gerards M, Mowbray C, Achilli A, Pyle A, Elson J, Howell N, La Morgia C (2007). Clinical expression of Leber hereditary optic neuropathy is affected by the mitochondrial DNA–haplogroup background. Am. J. Hum. Genet..

[31] Wang J, Peng L-Y, You C-P, Li Q-L, Wen M, Liu S-J, Hong Y-H (2014). Minifish mtDNA has abundance of repeat sequences and inefficient replication in vitro. CMM.

[32] Mi Z, Liu H, Zhang F (2020). Advances in the immunology and genetics of leprosy. Front. Immunol..

[33] Tió-Coma M, Kiełbasa SM, van den Eeden SJF, Mei H, Roy JC, Wallinga J, Khatun M, Soren S, Chowdhury AS, Alam K (2021). Blood RNA signature RISK4LEP predicts leprosy years before clinical onset. EBioMedicine.

[34] Rossmann MP, Dubois SM, Agarwal S, Zon LI (2021). Mitochondrial function in development and disease. Dis. Models Mech..

[35] Vanlalhruaii Tonsing M, VanlalbiakdikiSailo C, Zothansanga, Chhakchhuak L, Chhakchhuak Z, Pandit B, Kumar D, Pratim Mazumder P, Senthil Kumar N (2020). Analysis of variants in mitochondrial genome and their putative pathogenicity in tuberculosis patients from Mizoram, North east India. Mitochondrion.

[36] Sambrook J (1989). Molecular Cloning: A Laboratory Manual.

[37] Falk MJ, Shen L, Gonzalez M, Leipzig J, Lott MT, Stassen APM, Diroma MA, Navarro-Gomez D, Yeske P, Bai R (2015). Mitochondrial Disease Sequence Data Resource (MSeqDR): A global grass-roots consortium to facilitate deposition, curation, annotation, and integrated analysis of genomic data for the mitochondrial disease clinical and research communities. Mol. Genet. Metab..

[38] Karczewski KJ, Francioli LC, Tiao G, Cummings BB, Alföldi J, Wang Q, Collins RL, Laricchia KM, Ganna A, Birnbaum DP (2020). The mutational constraint spectrum quantified from variation in 141,456 humans. Nature.

[39] Sherry ST, Ward M, Sirotkin K (1999). dbSNP-database for single nucleotide polymorphisms and other classes of minor genetic variation. Genome Res..

[40] Landrum MJ, Lee JM, Benson M, Brown GR, Chao C, Chitipiralla S, Gu B, Hart J, Hoffman D, Jang W (2018). ClinVar: Improving access to variant interpretations and supporting evidence. Nucleic Acids Res..

[41] R Core Team. R: A language and environment for statistical computing (R Foundation for Statistical Computing, 2014).

[42] Team, J. JASP (Version 0.16. 3) [Computer software] (2022).

[43] Wickham H, Chang W, Wickham MH (2016). Package ‘ggplot2’. Create elegant data visualisations using the grammar of graphics. Version.

[44] Lex A, Gehlenborg N, Strobelt H, Vuillemot R, Pfister H (2014). UpSet: Visualization of intersecting sets. IEEE Trans. Vis. Comput. Graph..

